# Endometriosis classification systems: an international survey to map current knowledge and uptake †,‡

**DOI:** 10.52054/FVVO.14.1.001

**Published:** 2022-04-03

**Authors:** International Working Group of AAGL, ESGE, ESHRE and WES, K.T. Zondervan, S Missmer, M.S. Abrao, J.I. Einarsson, A.W. Horne, N.P. Johnson, T.T.M. Lee, J Petrozza, C Tomassetti, N Vermeulen, G Grimbizis, R.L. De Wilde

**Affiliations:** Nuffield Department of Women’s & Reproductive Health, University of Oxford, Oxford Endometriosis CaRe Centre, Oxford, Oxfordshire, UK; University of Oxford, Wellcome Centre for Human Genetics, Oxford, Oxfordshire, UK; Department of Obstetrics, Gynecology and Reproductive Biology, Michigan State University College of Human Medicine, East Lansing, MI, USA; Department of Epidemiology, Harvard University T H Chan School of Public Health, Boston, MA, USA; World Endometriosis Research Foundation, WERF, London, UK; Disciplina de Ginecologia, Departamento de Obstetricia e Ginecologia, Faculdade de Medicina FMUSP, Universidade de Sao Paulo, Sao Paulo, SP, Brazil; Gynecologic Division, BP-A Beneficencia Portuguesa de Sao Paulo, Sao Paulo, SP, Brazil; Division of Minimally Invasive Gynecologic Surgery, Department of Obstetrics and Gynecology, Brigham and Women’s Hospital, Boston, MA, USA; University of Edinburgh, MRC Centre for Reproductive Health, QMRI, Edinburgh, UK; Robinson Research Institute, University of Adelaide, Adelaide, SA, Australia; Department of Obstetrics, Gynecology and Reproductive Sciences, Magee Womens Hospital of UPMC, Pittsburgh, PA, USA; Department of Obstetrics and Gynecology, Massachusetts General Hospital Fertility Center, Boston, MA, USA; University Hospitals Leuven, Dept. Obstetrics and Gynaecology, Leuven University Fertility Center, Belgium; KU Leuven, Faculty of Medicine, Dept. Development and Regeneration, LEERM (Lab of Endometrium, Endometriosis and Reproductive Medicine), Belgium; ESHRE, Central Office, Strombeek-Bever, Belgium; 1st Dept Obstet Gynecol, Medical School, Aristotle University of Thessaloniki, Thessaloniki, Greece; Carl von Ossietzky Universitat Oldenburg, University Hospital for Gynecology, Oldenburg, Germany

**Keywords:** Endometriosis, infertility, classification, staging, reporting, survey, revised American Society for Reproductive Medicine, endometriosis fertility index, ENZIAN

## Abstract

**Background:**

In the field of endometriosis, several classification, staging and reporting systems have been developed and published, but there are no data on the uptake of these systems in clinical practice.

**Objectives:**

The objective of the current study was to examine whether clinicians routinely use the existing endometriosis classification systems, which system do they use and what are the clinicians’ motivations?

**Materials and Methods:**

A cross-sectional study was performed to gather data on the current use of endometriosis classification systems, problems encountered and interest in a new simple surgical descriptive system for endometriosis. Of particular focus were three systems most commonly used: the Revised American Society for Reproductive Medicine (rASRM) classification, the Endometriosis Fertility Index (EFI), and the ENZIAN classification. Data were analysed by SPSS. A survey was designed using the online SurveyMonkey tool consisting of 11 questions concerning three domains— participants’ background, existing classification systems and intentions with regards to a new classification system for endometriosis. Replies were collected between 15 May and 1 July 2020.

**Main outcome measures:**

Uptake, feedback and future intentions.

**Results:**

The final dataset included the replies of 1178 clinicians, including surgeons, gynaecologists, reproductive endocrinologists, fertility specialists and sonographers, all managing women with endometriosis in their clinical practice. Overall, 75.5% of the professionals indicate that they currently use a classification system for endometriosis. The rASRM classification system was the best known and used system, the EFI system and ENZIAN system were known by a majority of the professionals but used by only a minority. The lack of clinical relevance was most often selected as a problem with using any system. The findings of the survey suggest that clinicians worldwide are open to using a new classification system for endometriosis that can achieve standardised reporting, and is clinically relevant and simple.

**Conclusions:**

Even with a high uptake of the existing endometriosis classification systems (rASRM, ENZIAN and EFI), most clinicians managing endometriosis would like a new simple surgical descriptive system for endometriosis.

**What is new?:**

The findings therefore support future initiatives for the development of a new descriptive system for endometriosis and provide information on user expectations and conditions for universal uptake of such a system.

## Introduction

In the field of endometriosis, several classifications, staging and descriptive systems have been developed, however, none seem to be comprehensive, or correlate sufficiently with clinically relevant outcomes for general uptake. In an attempt to provide direction for the future development of a new endometriosis classification system that is clinically relevant, three essential projects were defined: to review existing classification and staging systems for endometriosis; to develop a standard glossary to be utilised across the field of endometriosis; and to assess the current knowledge and uptake of classification systems among practitioners in the field.

In the first project, 22 published classification and staging systems for endometriosis were summarised as well as the studies evaluating these with regards to feasibility, validity and reproducibility (International Working Group of AAGL ESGE ESHRE and WES, et al., 2021d, e, f). The second project resulted in the publication of a terminology for endometriosis (International Working Group of AAGL ESGE ESHRE and WES et al., 2021a, b, c). For the third project, considering the uptake of the different classification systems, we conducted a survey to find out whether clinicians were routinely using any classification for endometriosis in clinical practice, which system is used most frequently, and what the motivations of clinicians are to use, or not use, any classification in endometriosis.

The current paper reports the results of the survey. Of particular focus were the three systems most commonly used: the Revised American Society for Reproductive Medicine (rASRM) classification (American Society for Reproductive Medicine, 1997), the endometriosis fertility index (EFI) (Adamson and Pasta, 2010), and the ENZIAN classification (Tuttlies et al., 2005). With regards to the ENZIAN classification, a revised version of the classification, #ENZIAN (Keckstein et al., 2021), has meanwhile been published, but this was not available and hence not considered when the survey was conducted.

## Materials and Methods

A cross-sectional study was conducted using an online survey, which focused on classification of endometriosis. The questions were drafted by an international group of experts in endometriosis representing four societies: the American Association of Gynecologic Laparoscopists (AAGL), European Society for Gynaecological Endoscopy (ESGE), ESHRE, and the World Endometriosis Society (WES). The survey was conducted online and afterwards distributed amongst all members of the participating societies and the members of the American Society for Reproductive Medicine (ASRM).

The survey included 11 questions organised in three sections. The first section focused on the participants’ background and included questions related to their country, professional status (profession, experience) and expertise in managing endometriosis patients (Supplementary Data 1). The second part of the survey focused on existing classification systems, and the third part on the uptake of a potential new descriptive system for endometriosis.

The survey was open between 15 May and 1 July 2020. Recruitment strategies included mass mailings by each of the participating societies and promotion on social media. A total of 1251 replies were received.

The results of the survey were exported to SPSS 19 (IBM Corp, Armonk, NY, USA) for Windows for further analysis. Analysis and comparisons were focused on respondents who treat patients with endometriosis in clinical practice. Two sub- analyses were conducted, comparing surgeons versus other physicians, and replies between different regions. Statistically significant differences (p<0.05) were assessed through Chi- square analysis.

## Results

**Figure 1:**
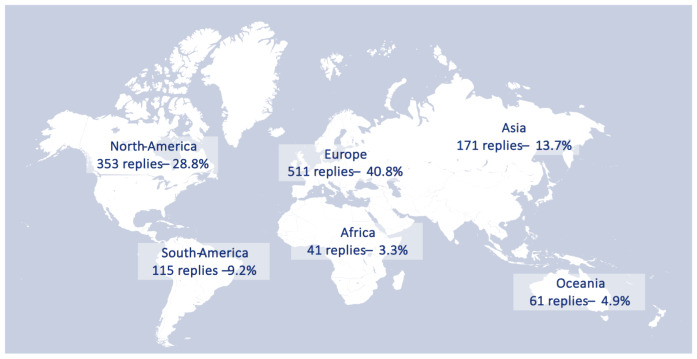
Overview of replies to the survey on endometriosis classification systems, across different regions.

**Figure 2:**
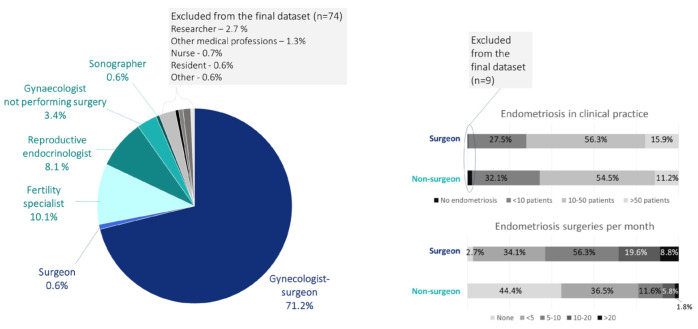
Overview of professions and expertise of the respondents.

Of the 1251 respondents to the survey, the majority represented Europe (40.8%) and North-America (28.8%) (Fig 1). Figure 2 shows the frequencies of the profession of the respondents. For the final analysis, responders were restricted to practicing clinicians, which included non-gynaecologist surgeons and gynaecologist-surgeon ("surgeon"), and gynaecologists not performing surgery, reproductive endocrinologists, fertility specialists and sonographers ("non-surgeon"). From these groups, nine respondents were excluded as they reported they did not manage women with endometriosis in their clinical practice. The final dataset included 1178 respondents. One-third of these reported managing less than 10 endometriosis patients per month, and this proportion did not differ between the surgeon and non-surgeon groups. Within the surgeon group, 85% reported performing more than ve endometriosis surgeries per month (Fig. 2).

### Knowledge and use of existing classification systems

**Figure 3:**
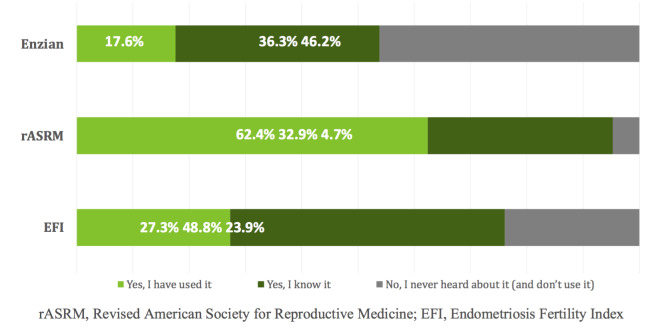
Knowledge and use of ENZIAN, rASRM and EFI.

The rASRM classification system was the best known and most frequently used system, with only 4.7% of the respondents indicating they did not know or use the system. The EFI system and ENZIAN system were known by 76.1% and 53.8% of respondents, respectively, but used by only a minority (27.3% for EFI, 17.6% for ENZIAN) (Fig. 3).

**Figure 4:**
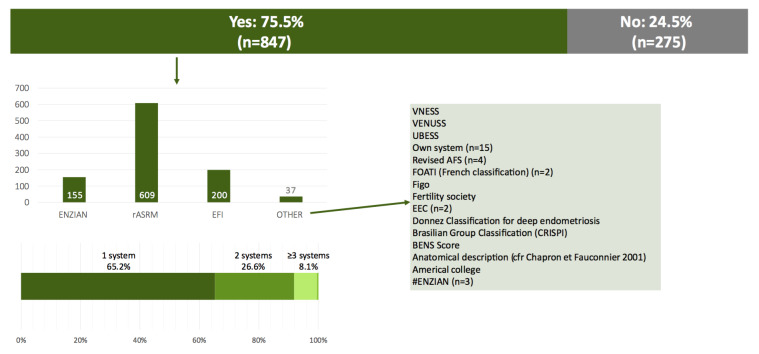
Current use of a classification system, and which system is used.

Overall, 75.5% of the respondents indicated that they currently use a classification system for endometriosis. One-third of the respondents further reported that they use more than one system (26.6% uses two systems, 8.1% uses three or more systems). The rASRM system was most often used. A minority of respondents (3.7%, n=37) indicated that they use another published classification system (not ENZIAN, rASRM or EFI) or their own system (Fig. 4).

**Figure 5:**
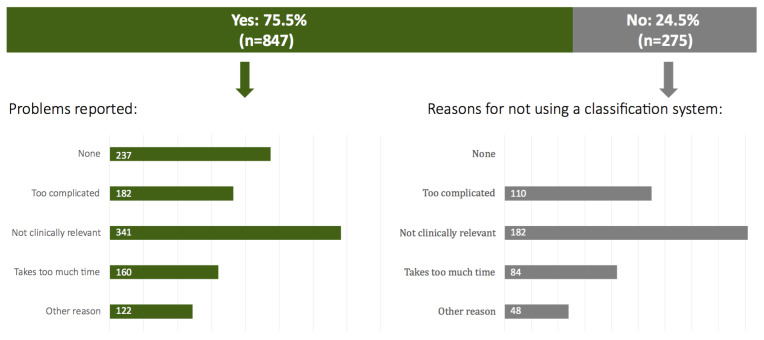
Problems with use of the existing classification systems and reasons for not using a classification system.

On the question of which problems responders had encountered with the existing classification systems, 22.7% replied that they do not encounter any problems. The remaining respondents indicated a variety of problems. The lack of clinical relevance (n=341) was most often selected (Fig. 5).

### Motivation to use a new simple surgical descriptive system for endometriosis

**Figure 6:**
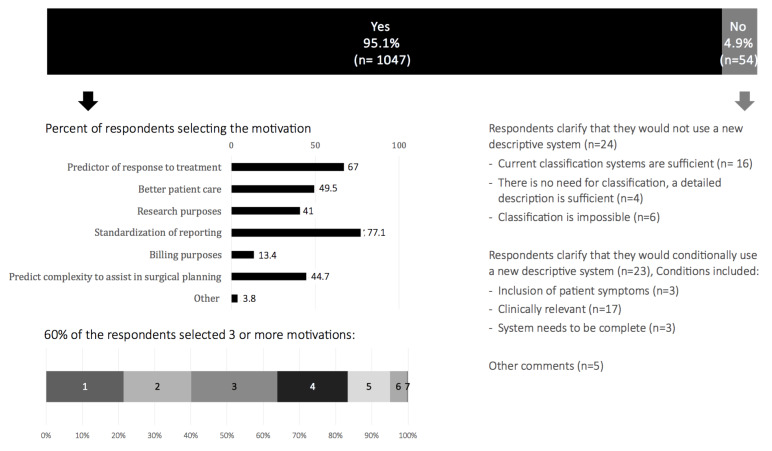
Interest in a new simple surgical descriptive system for endometriosis.

The vast majority of respondents (95.1%) replied positive to the question on whether they would use a simple surgical descriptive system for endometriosis, if available (Fig. 6). They indicated that standardisation of reporting and prediction of response to treatment would be the main motivating factors to do so. Of the 4.9% of respondents not motivated to use a new system, some explained they were happy with the existing systems, while others considered that classification in endometriosis was not needed or impossible. The rest of respondents would use the system if it included patient symptoms, was clinically relevant and/or complete.

### Surgeon versus non-surgeon

The responses were compared between those respondents the indicated surgeon (non-gynaecologists) or gynaecologist-surgeon as their profession, and other clinicians (gynaecologists not performing surgery, reproductive endocrinologists, fertility specialists, sonographers) (Table I). There were no clear differences between surgeons and non-surgeons with regards to the knowledge and use of any classification systems, although surgeons more often reported using the ENZIAN classification (25.5% versus 7.7%, p= .00001). With regards to the reasons for not using a classification system, surgeons more often indicated the lack of clinical relevance compared to non-surgeons (75.0% versus 51.7%, p= .00058). With regards to a new descriptive system, surgeons more frequently reported the following motivations, compared to non-surgeons: to predict complexity to assist in surgical planning (50.4% versus 25.0%, p< 0.00001), billing purposes (15.8% versus 5.2%, p= .000017), standardisation of reporting (79.6% versus 68.5%, p= .00026), and research purposes (42.9% versus 34.3%, p=.014).

**Table I:** Comparison of the replies of surgeons versus non-surgeons to questions in a survey on use of endometriosis classification systems.

		SURGEON†		NON-SURGEON††		Chi-square
		N	%	N	%	
DEMOGRAPHICS						
Continent	Africa	25	2.8%	14	5.2%	P=0.004
	Asia	118	13.1%	45	16.6%	
	Europe	377	42.0%	101	37.3%	
	North-America	244	27.2%	73	26.9%	
	Oceania	53	5.9%	4	1.5%	
	South-America	81	9.0%	34	12.5%	
Endometriosis in clinical practice (n=1169)	None	0	0	0	0	P=0.091
	< 10 patients per month	248	27.6%	89	32.8%	
	10 - 50 patients per month	507	56.5%	151	55.7%	
	> 50 patients per month	143	15.9%	31	11.4%	
Endometriosis surgeries per month (n=1169)	None	21	2.3%	118	43.5%	P<0.001
	<5	307	34.2%	100	36.9%	
	5-10	314	35.0%	32	11.8%	
	10-20	177	19.7%	16	5.9%	
	>20	79	8.8%	5	1.8%	
KNOWLEDGE AND USE OF EXISTING SYSTEMS						
Enzian (n=1122)	Knowledge	322	37.2%	85	33.2%	P=0.005
	Use	165	19.1%	32	12.5%	
	No knowledge/use	379	43.8%	139	54.3%	
Revised ASRM system (n=1122)	Knowledge	285	32.9%	84	32.8%	P=0.69
	Use	543	62.7%	157	61.3%	
	No knowledge/use	38	4.4%	15	5.9%	
Endometriosis Fertility Index (EFI) (n=1122)	Knowledge	416	48.0%	132	51.6%	P=0.31
	Use	234	27.0%	72	28.1%	
	No knowledge/use	216	24.9%	52	20.3%	
Current use of any classification system (n=1122)	Yes	650	75.1%	197	77.0%	P=0.54
	No	216	24.9%	59	23.0%	
Use of classification system	Total	568		130		P<0.001
	Enzian*	145	25.5%	10	7.7%	
	rASRM	492	86.6%	117	90.0%	
	EFI	158	27.8%	42	32.3%	
	Other	34	6.0%	3	2.3%	
Problems with current classification systems	Total	642		192		P=0.15
	None	181	28.2%	56	29.2%	
	Too complicated*	151	23.5%	31	16.1%	
	Not clinically relevant	273	42.5%	68	35.4%	
	Takes too much time	124	19.3%	36	18.8%	
	Other	92	14.3%	30	15.6%	
Reasons for not using a classification system	Total	216		58		P<0.001
	Existing systems are too complicated	90	41.7%	20	34.5%	
	Existing systems are not clinically relevant*	162	75.0%	30	51.7%	
	Existing systems take too much time to complete	69	31.9%	15	25.9%	
	Other reason	31	14.4%	17	29.3%	
NEW DESCRIPTIVE SYSTEM						
Interested in use of a simple surgical descriptive system (n=1101)	Yes	816	95.7%	231	93.1%	P=0.11
	No	37	4.3%	17	6.9%	
Primary motivation to use a descriptive system?	Total	853		248		P<0.001
	Predictor of response to treatment	563	66.0%	175	70.6%	
	Better patient care	422	49.5%	123	49.6%	
	Research purposes*	366	42.9%	85	34.3%	
	Standardization of reporting*	679	79.6%	170	68.5%	
	Billing purposes*	135	15.8%	13	5.2%	
	Predict complexity to assist in surgical planning*	430	50.4%	62	25.0%	
	Other	32	3.8%	10	4.0%	

† non-gynaecologist and gynaecologist surgeons; †† gynaecologists not performing surgery, reproductive endocrinologists, fertility specialists, sonographers; *Significant (p<0.05) in comparing surgeons versus non-surgeons ; rASRM: Revised American Society for Reproductive Medicine.

### Differences between regions

In the comparison by continent, there was significant variation in the frequency of professions of the respondents and consequently in the number of surgeries they performed (Table II), but the level of expertise with endometriosis (i.e. the number of patients seen in clinical practice) was similar. Across continents, between 73.5% and 80.4% of respondents stated they currently use a classification system. There was lower knowledge and use in North-America, as compared to the rest of the world concerning ENZIAN (32.0% versus 62.1%, p<0.00001) and EFI (60.5% versus 82.0%, p<0.00001).

**Table II:** Comparison of replies to the survey by continent.

		Africa		Asia		Europe		North- America		Oceania		South-America	
		N	%	N	%	N	%	N	%	N	%	Ns	%
DEMOGRAPHICS						
Profession	Surgeon (non- gynaecologist)	0	0.0%	2	1.2%	3	0.6%	2	0.6%	0	0.0%	1	0.9%
	Gynaecologist-surgeon	25	64.1%	116	71.2%	374	78.2%	242	76.3%	53	93.0%	80	69.6%
	Gynaecologist not performing surgery	0	0.0%	2	1.2%	29	6.1%	3	0.9%	2	3.5%	5	4.3%
	Reproductive endo-crinologist	3	7.7%	12	7.4%	23	4.8%	55	17.4%	1	1.8%	5	4.3%
	Fertility specialist	11	28.2%	31	19.0%	47	9.8%	14	4.4%	1	1.8%	20	17.4%
	Sonographer	0	0.0%	0	0.0%	2	0.4%	1	0.3%	0	0.0%	4	3.5%
Endometriosis in clinical practice (n=1169)	< 10 patients per month	18	46.2%	52	31.9%	124	25.9%	99	31.2%	9	15.8%	35	30.4%
	10 - 50 patients per month	17	43.6%	88	54.0%	281	58.8%	175	55.2%	38	66.7%	59	51.3%
	> 50 patients per month	4	10.3%	23	14.1%	73	15.3%	43	13.6%	10	17.5%	21	18.3%
Endometriosis surgeries per month (n=1169)	None	8	20.5%	21	12.9%	65	13.6%	22	6.9%	3	5.3%	20	17.4%
	< 5	14	35.9%	66	40.5%	148	31.0%	128	40.4%	11	19.3%	40	34.8%
	5-10	10	25.6%	50	30.7%	145	30.3%	94	29.7%	17	29.8%	30	26.1%
	10-20	6	15.4%	16	9.8%	77	16.1%	51	16.1%	23	40.4%	20	17.4%
	>20	1	2.6%	10	6.1%	43	9.0%	22	6.9%	3	5.3%	5	4.3%
KNOWLEDGE AND USE OF EXISTING SYSTEMS						
Enzian (n=1122)	Knowledge	14	35.9%	55	35.3%	186	41.0%	76	24.6%	20	35.7%	56	51.9%
	Use	6	15.4%	16	10.3%	130	28.6%	23	7.4%	4	7.1%	18	16.7%
	No knowledge/ use	19	48.7%	85	54.5%	138	30.4%	210	68.0%	32	57.1%	34	31.5%
Revised ASRM system (n=1122)	Knowledge	15	38.5%	64	41.0%	146	32.2%	100	32.4%	10	17.9%	34	31.5%
	Use	20	51.3%	85	54.5%	282	62.1%	200	64.7%	44	78.6%	69	63.9%
	No knowledge/ use	4	10.3%	7	4.5%	26	5.7%	9	2.9%	2	3.6%	5	4.6%
Endometriosis Fertility Index (EFI) (n=1122)	Knowledge	20	51.3%	81	51.9%	239	52.6%	125	40.5%	23	41.1%	60	55.6%
	Use	14	35.9%	37	23.7%	136	30.0%	62	20.1%	21	37.5%	36	33.3%
	No knowledge/ use	5	12.8%	38	24.4%	79	17.4%	122	39.5%	12	21.4%	12	11.1%
Current use of any classification system (n=1122)	Yes	29	74.4%	117	75.0%	343	75.6%	227	73.5%	45	80.4%	86	79.6%
	No	10	25.6%	39	25.0%	111	24.4%	82	26.5%	11	19.6%	22	20.4%
Use of classification system	Total	21		88		267		210		41		71	
	Enzian	2	9.5%	12	13.6%	113	42.3%	12	5.7%	0	0.0%	16	22.5%
	rASRM	18	85.7%	75	85.2%	226	84.6%	195	92.9%	38	92.7%	57	80.3%
	EFI	6	28.6%	32	36.4%	90	33.7%	35	16.7%	10	24.4%	27	38.0%
	Other	0	0.0%	1	1.1%	19	7.1%	8	3.8%	6	14.6%	3	4.2%
Problems with current classification systems	Total	28		115		335		226		45		85	
	None	8	28.6%	30	26.1%	105	31.3%	66	29.2%	11	24.4%	17	20.0%
	Too complicated	4	14.3%	30	26.1%	60	17.9%	56	24.8%	16	35.6%	16	18.8%
	Not clinically relevant	10	35.7%	49	42.6%	121	36.1%	105	46.5%	25	55.6%	31	36.5%
	Takes too much time	7	25.0%	23	20.0%	52	15.5%	47	20.8%	10	22.2%	21	24.7%
	Other reason	3	10.7%	9	7.8%	58	17.3%	31	13.7%	6	13.3%	15	17.6%
Reasons for not using a classification system	Total	10		39		110		82		11		22	
	Existing systems are too complicated	4	40.0%	15	38.5%	49	44.5%	31	37.8%	6	54.5%	5	22.7%
	Existing systems are not clinically relevant	4	40.0%	21	53.8%	74	67.3%	72	87.8%	7	63.6%	14	63.6%
	Existing systems take too much time to complete	6	60.0%	13	33.3%	35	31.8%	24	29.3%	1	9.1%	5	22.7%
	Other reason	2	20.0%	6	15.4%	20	18.2%	13	15.9%	1	9.1%	6	27.3%
NEW DESCRIPTIVE SYSTEM						
Interested in use of a simple surgical descriptive system (n=1101)	Yes	37	97.4%	151	98.1%	413	94.3%	290	94.2%	50	89.3%	106	99.1%
	No	1	2.6%	3	1.9%	25	5.7%	18	5.8%	6	10.7%	1	0.9%
Primary motivation to use a descriptive system?	Total	38		154		438		308		56		107	
	Predictor of response to treatment	25	65.8%	116	75.3%	277	63.2%	208	67.5%	31	55.4%	81	75.7%
	Better patient care	17	44.7%	84	54.5%	208	47.5%	163	52.9%	30	53.6%	43	40.2%
	Research purposes	9	23.7%	52	33.8%	185	42.2%	145	47.1%	26	46.4%	34	31.8%
	Standardization of reporting	32	84.2%	102	66.2%	350	79.9%	242	78.6%	46	82.1%	77	72.0%
	Billing purposes	4	10.5%	6	3.9%	20	4.6%	102	33.1%	7	12.5%	9	8.4%
	Predict complexity to assist in surgical planning	13	34.2%	56	36.4%	186	42.5%	172	55.8%	20	35.7%	45	42.1%
	Other	0	0.0%	5	3.2%	19	4.3%	11	3.6%	2	3.6%	5	4.7%

With regards to the primary motivation to use a descriptive system, standardisation was most often selected in all continents, apart from Asia and South-America, where prediction of response to treatment was the primary motivation. These results, specifically for Oceania and Africa, should be considered with caution considering the low number of replies from these areas.

## Discussion

This report summarises the replies of 1178 clinicians, including surgeons, gynaecologists, reproductive endocrinologists, fertility specialists and sonographers, all managing women with endometriosis in their clinical practice. Questions focused on the current use of endometriosis classification systems, problems encountered and interest in a new simple surgical descriptive system for endometriosis.

Overall, three-quarters of the respondents indicate that they use a classification system for endometriosis, with limited variation according to profession or location. The rASRM classification system, the oldest system, was the best known and used. The ENZIAN classification system, published in 2005, and the EFI system, published in 2010, were known by half of the respondents, but used less often, by 1 in 5 and 1 in 4 clinicians, respectively. The ENZIAN classification system was more often used by surgeons.

Our results highlight some problems with the currently available classification systems. The most often reported problem, both by physicians using a classification system and those that do not, is the lack of clinical relevance. The complexity of the currently available classification systems is also considered a barrier for uptake, which is in line with previous reports (Adamson, 2011, Johnson et al., 2017). It should be noted, in this respect, that the results of the present survey reflect the ENZIAN classification, and can not necessarily be extrapolated to the revised version of the classification, #ENZIAN (Keckstein et al., 2021).

In contrast to the high uptake of the rASRM, ENZIAN and EFI systems, the vast majority of clinicians managing endometriosis intended to use a new simple surgical descriptive system for endometriosis if developed. Standardisation of reporting and prediction of response to treatment would be the main motivating factors to do so: the latter is consistent with the lack of clinical relevance of the current available systems. Standardised reporting of surgical findings is implemented in the WERF EPHect (Becker et al., 2014) and CORDES (Vanhie et al., 2016) questionnaires, which are currently tools for research purposes and not intended for clinical reporting. Any new clinically relevant classification system would need to be designed based on robust data analysis, by a multidisciplinary team, including experts in classification system development, and validated across settings for its intended utility. Currently, the EFI is the only classification for which such testing was conducted in multiple studies in different countries. It is vital that both design and validation studies of any new tool would require robust assessment of metrics such as association with patient outcomes, including prediction of response to treatment, if the tool is intended for this clinical purpose. A standardised reporting system and anatomical classification of the endometriosis findings is a necessity for the further development of a grading system for clinical prediction.

Although confined to the inherent limits of the methodology, this report provides relevant information with regards to the uptake of currently available systems and suggests that clinicians worldwide are open to using a new classification system for endometriosis that can achieve standardised reporting, and is clinically relevant and simple. These considerations should be taken into account in the development of future endometriosis classifications.

## What does this mean for patients?

Classification systems for endometriosis have been developed, but there is no data as to whether clinicians actually use them in the management of their patients in clinical practice. We have organised a large survey to gather this information, and we found that indeed a large number of clinicians use the existing classification systems. The clinicians also mentioned a number of issues with the existing classification systems, including that the systems may not be very relevant for the diagnosis or treatment of the patient, or that they are not linked to the patients’ symptoms. Finally, the clinicians answering the survey made suggestions on how to improve the classification systems. The information collected is very valuable towards future updates of existing classification systems, or development of a new, simple and clinically relevant universal system.
